# THE IMPACTS OF CHANGES IN DIABETES AND ORAL HEALTH CONDITIONS ON COGNITIVE TRAJECTORIES

**DOI:** 10.1093/geroni/igac059.1097

**Published:** 2022-12-20

**Authors:** Bei Wu, Chenxin Tan, Huabin Luo, Xiang Qi

**Affiliations:** New York University, New York, New York, United States; New York University, New York, New York, United States; East Carolina University, Greenville, North Carolina, United States; New York University, New York City, New York, United States

## Abstract

Despite the emerging research studying the relationship between diabetes mellitus (DM), oral health problems, and cognitive function, little is known about how changes in DM and oral health status affected the trajectories of cognitive decline. Using data of 12,802 participants aged 51+ from the 2006-2018 English Longitudinal Study of Ageing, this study examined the longitudinal relationships of cognitive functions with time-varying DM and oral health status – measured by edentulism, self-rated dental condition, and oral impact on daily performance (OIDP) scale at five time-points from 2006 to 2018. Results showed that participants had DM and edentulism throughout the study period had an accelerated decline in verbal fluency (mean=20.9; b=-0.17, 95% CI=-0.28, -0.07); and those had both DM and OIDP-problem had an accelerated decline in memory function (mean=10.4; b=-0.09, 95% CI=-0.15, -0.03). Prevention and treatment of multiple chronic conditions are essential for cognitive health in later life.

